# Characterization of Microstructural Changes on Biglycan Induced Mice Bone by Low-Field Nuclear Magnetic Resonance

**DOI:** 10.31058/j.ap.2021.42004

**Published:** 2021-06-03

**Authors:** Qingwen Ni, Rui Hua, Douglas Holland, Anahi Tinajero, Yan Han, Jean X. Jiang, Xiaodu Wang

**Affiliations:** 1 Department of Mathematics and Physics,Texas A&M International University, Laredo, TX, USA; 2 Department of Mechanical Engineering, The University of Texas at San Antonio, San Antonio, TX, USA; 3 Department of Biochemistry and Structural Biology, The University of Texas Health Science Center, San Antonio, TX, USA

**Keywords:** NMR, Mouse Bone, Bound Water, Biglycan

## Abstract

A NMR spin-spin (T_2_) relaxation technique has been described for determining the porosity, and the bound water distribution in biglycan induced mouse bone and correlate to their mechanical properties. The technique of low-field proton NMR involves spin-spin relaxation and free induction decay (FID) measurements, and the computational inversion methods for decay data analysis. The CPMG T2 relaxation data can be inverted to T_2_ relaxation distribution and this distribution then can be transformed to a pore size distribution with the longer relaxation times corresponding to larger pores. The FID T_2_ relaxation data of dried bone (mobile water removed) can be inverted to T_2_ relaxation distribution and this distribution then can be transformed to bound and solid-like water distribution with the longest relaxation time corresponding to bound water component. These techniques are applied to quantify apparent changes in porosity, and bound water in controlled and biglycan knockout mouse bone. Overall bone porosity from CPMG T_2_ relaxation is determined using the calibrated NMR fluid volume from the proton relaxation data divided by overall bone volume. Ignore the physical sample differences, from the inversion FID T_2_ relaxation spectrum, the ratio of the bound to solid-like water components is used to calibrate the bound water inside bone, and the results can be used to correlated bone mechanical properties. Hydration status significantly affects the toughness of bone, and bound water has been considered as a biomarker for prediction of bone fragility fractures. In addition to the collagen phase, recent evidence shows that glycosaminoglycans (GAGs) of proteoglycans (PGs) in the extracellular matrix also play a pivotal role in regulating the tissue-level hydration status of bone, there by affecting the tissue-level toughness of bone. Furthermore, biglycan and decorin are two major types of PGs in bone reports. Biglycan knockout induced changes in GAGs, bound water, as well as bone tissue toughness. Among all subtypes of PGs, biglycan is identified as a major subtype in the bone mineral matrix. In this study, we used a biglycan mouse model and the obtained bone samples were measured by low-field NMR to determine the bone porosity and bound water changes, and used to predict if knockout of biglycan may affect the amount of bound water and subsequently lead to reduce toughness of bone.

## 1. Introduction

Previous study has revealed that water in bone is present in three different conformations; namely freely mobile water in pores; bound water at surfaces and/or within the mineral and collagen phases; and structural water as part of collagen and mineral molecules [1,2]. In addition, evidence shows that water may also play a major role in viscous response of bone [3], most likely *via* the so-called sacrificial bonding mechanism [4]. In fact, hydration status significantly affects the toughness of bone, and bound water has been considered as a biomarker for prediction of bone fragility fractures [5,6,7]. In addition to the collagen phase, recent evidence shows that glycosaminoglycans (GAGs) of proteoglycans (PGs) in the extracellular matrix also play a pivotal role in regulating the tissue-level hydration status of bone, there by affecting the tissue-level toughness of bone. And biglycan and decorin are two major types of PGs in bone reports. Biglycan knockout induced changes in GAGs, bound water, as well as bone tissue toughness. Among all subtypes of PGs, biglycan is identified as a major subtype in the bone mineral matrix [8,9,10]. In this study, we used a biglycan mouse model and the obtained bone samples were measured by low-field NMR to determine the bone porosity and bound water changes, and used to predict if knockout of biglycan may affect the amount of bound water and subsequently lead to reduce toughness of bone.

Here, the techniques of low-field pulsed proton nuclear magnetic resonance (NMR) spin relaxation and in addition with high-field NMR are described for assessment of structural changes of normal and disuse (biglycan knockout) mice bone in situ. It is known that the total amplitude of T_2_ relaxation envelopes, measured by the NMR spin echo train (CPMG) [11,12], is a representation of the liquid phase inside the pores. Therefore, the NMR CPMG magnetization amplitude can be transferred to the volume of water after calibration with the NMR signal amplitude of the known volume of the selected water. In this study, the distribution of mobile water, porosity that can be determined by using low-field (20 MHz) CPMG relaxation technique, and the pore size distributions can be determined by a computational inversion relaxation method. It is also known that the total proton intensity of magnetization from the NMR free induction decay (FID) signal is due to the water present inside the pores (mobile water), the water that has undergone hydration with the bone (bound water), and the protons in the collagen and mineral matter (solid-like protons) [13]. Therefore, the components of total solid and bound water within bone that can be determined by low-field NMR free induction decay technique. This technique involves spin-spin relaxation measurement and inversion spin-spin relaxation spectral analysis methods.

## 2. Materials and Methods

### 2.1. Sample Preparation

A total of 14 mouse leg bone specimens with 7 in control and 7 in disuse (biglycan knockout) were obtained from 6 month-old male. It was used a biglycan, decorin, and biglycan/decorin deficient mouse models [14] to prepare the samples. Since biglycan (*Bgn*) gene is located at X chromosome, we crossed homozygous male (*Bgn*-/0) with heterozygous female (*Bgn*−/+) mice to generate homozygous (*Bgn*-/0) and WT (wild type) (*Bgn*+/0) littermate mice. The animals were sacrificed at the age of 6 months and the bone soft tissues were dissected and stored in −80 °C; and prior to NMR measurements, the samples were completely thawed at room temperature.

### 2.2. Low-Field NMR Measurement

A low-field NMR spectrometer (Bruker 20 MHz) was set up at a proton frequency of 20 MHz for these measurements. ^1^H spin-spin (T_2_) relaxation profiles were obtained by using NMR CPMG {90° [-τ- 180° – τ- (echo)]_n_ – T_R_} spin echo method with a 6.5 s wide 90° pulse, τ of 500 μs for mouse bone and T_R_ (sequences repetition rate) of 15 s. Each T_2_ profile, one thousand echoes (one scan with n = 1000) were acquired and sixty-four scans were used. Thus, one scan will have repeated 1000 echoes in the window. For each FID profile, 1000 data points were acquired in one scan (an approximate 2 ms delay window). The data was measured on fresh frozen leg tissues after complete thawing in the room temperature (21 ± 1°C). The full saturated bone tissues were used for CPMG measurements, and free air dried (mobile water removed) bone tissues were used for FID measurements.

### 2.3. High-Field NMR with Magic Angle Spinning (MAS) Measurement

High-Field proton NMR spectra were acquired on a Brucker 400 MHz superconducting solenoid magnet. For MAS experiments the rotor axis makes an angle of 54°44’, the “magic angle”, with the static applied field. For single pulse experiment, a 4.5 us pulse produced a 90° tip angle in MAS probe with spinning rate at 8 kHz. In order to reach this high spinning rate, the mice bone was put into the rotor first, then added Teflon powders to fill the empty space of the rotor to make the spinning uniformly. Only 16 scans were accumulated with quadrature phase cycling. The acquisition time was 4.0 s with 8 kHz sweep width. The bone tissues were free dried on air for 48 hours to remove the mobile water and used for measurements.

### 2.4. The Relationship between NMR Data and Effective Pore Sizes

Based on the low field NMR principle the diffusion effect may be negligible. Here, we accept the Brownstein and Tarr [15] assumption that the relaxation rate 1/T_2_ is proportional to the surface-to-volume (S/V) ratio of the pore

(1) $$1∕T_{2}=\rho (S∕V{)}_{pore}$$

where ρ is the surface relaxivity, which is a measure of the effects of the pore surface enhancing the relaxation rate [16]. Equation (1) indicates that the NMR relaxation time is proportional to pore size with longer relaxations corresponding to larger pores. For a porous bone, the observed NMR magnetization will depend upon the T_2_ of broad distributions of water in all pores. This implies that NMR transverse relaxation (T_2_) data can be expressed as a sum of exponential functions:

(2) $$M(t_{i})=f(T_{2,j})exp(\text{-}t_{i}∕T_{2,j})$$

where f(T_2,j_ )is proportional to the number of spins which relax with a time constant T_2,j_. M(t_i_) is the NMR magnetization decay from fluid saturated bone. Equation (2) can be inverted into a T_2_ relaxation time distribution. Thus, instead of estimating a single relaxation time from a magnetization decay, it is necessary to estimate an inversion T_2_ spectrum or distribution of relaxation time f(T_2,j_), and an inversion relaxation technique was applied [17,13]. Since T_2_ depends linearly upon pore size, the T2 distribution corresponds to pore-size distribution with the longer relaxation times having the larger pores. The method for the inversion of FID (T_2-Fid_) is similar to the inversion of CPMG data by using (echo) shorter as 1 μs and 2 ms for delay time in the measurements. Three separate relaxation components from inversion T_2-FID_ were detected corresponding to protons in solid, bound and mobile phases [13].

### 2.5. Bound Water Intensity Determined from NMR FID T2 Inversion Spectrum

From FID measurement for full saturated bone sample, after T_2_ inversion three peaks should be displayed that are (from left to right) [13] solid-like water component, bound water component, and liquid-like component. Since the liquid-like component intensity is too strong, and is not the important point at this moment (since the CPMG method already determine the detailed mobile water distributions), this time we used dried samples (the free water inside pore is removed by free air dry for 48 hours). Therefore, only two peaks (bound and solid-like) are displayed in inversion T_2_ relaxation spectrum (Figure 2). We are cognizant of the sample size variation, but known (after test) the solid-like intensity is proportional to the sample mass, then we propose the bound water changes with the sample by using the ratio of bound water component to solid-like component from inversion T_2_ relaxation spectrum.

## 3. Results and Discussions

**Figure 1.**
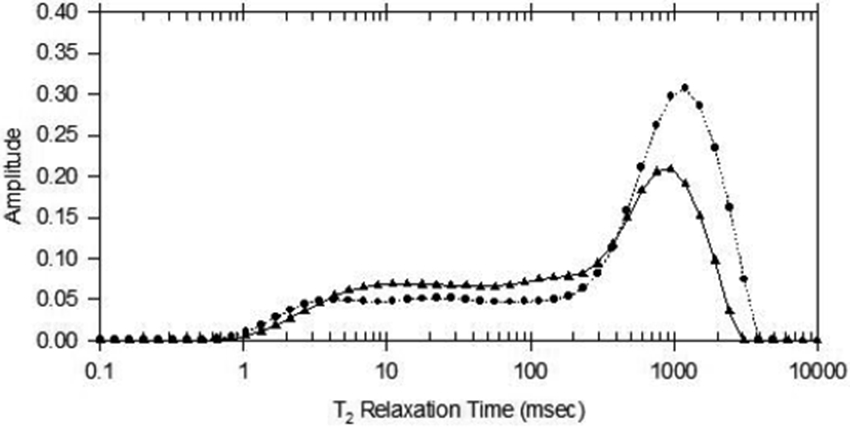
Inversion CPMG T_2_ relaxation time spectra for mouse WT and KO leg bones. The high intensity is for KO (•), with porosity 37.7%, and the low intensity (▲) is for WT, with porosity 21.33%, respectively, measured by low-field NMR.

Comparison of disuse mouse leg bone group with control leg bone group, the significant differences are found. For example, Figure 1 shows the inversion NMR CPMG relaxation data spectra. The porosity, and the pore size differences for sample #5 between the control WT and the disuse KO are clearly observed. In Figure 1, the high intensity one is for KO (·), and low intensity one is for WT (▲). Since the longer relaxation times are corresponding to larger pores, and the higher intensity corresponding to higher porosity, therefore, KO one has larger pores and porosity than the WT one. The comparison of porosities between the KO and WT groups are listed on Table 1.

**Figure 2.**
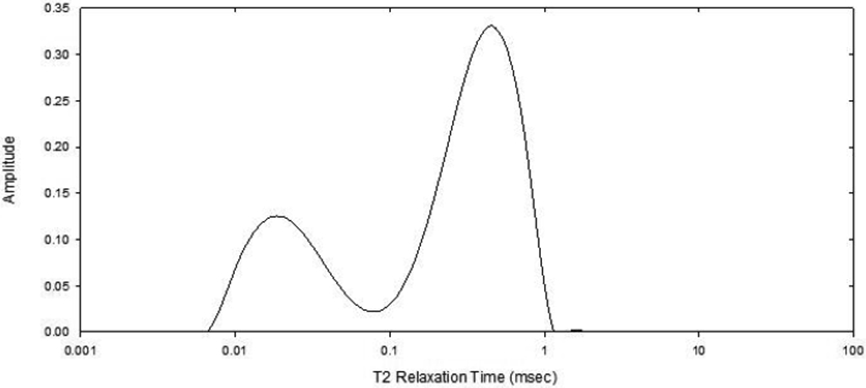
Example of inversion FID T2 relaxation time spectra for mouse, measured by low-field NMR. The peaks from left to right are solid-like water and bound water, respectively.

Figure 2 shows the example of inversion FID spectrum, after the mobile water was removed by free air dry. It is because in mice bone tissues the mobile water single is overwhelming than the bound water single, and the mobile water was not the interesting at this moment. Therefore, only two water peaks are observed with left first is solid-like component and second is bound water component. From low-field NMR measurement, the relative amount of bound water was then estimated as the ratio of the total intensity of bound water signal with respect to the total intensity of the solid-like water signal (representative of bone mass because in these sample measurements, the solid form proton signal intensities were correlated with the sample mass weights) of each sample. It is known that the T_2_ relaxation distributions for liquid-like phase are from milliseconds to seconds (Figure 1), while the T_2_-Fid relaxation time distributions are only from microseconds to milliseconds even for three different phase components [13]. The major difference between the T_2_ relaxation times obtained using the NMR FID and CPMG methods is that the T_2_ relaxation time constant is significantly shorter from FID technique than in CPMG technique. This is because that in FID measurement the signal decays in a time T_2_-Fid that often is determined by field inhomogeneity while an ingenious CPMG measurement is a practical method for overcoming the inhomogeneity problem [18].

The estimated results for ratio of the bound water to solid-like water were shown in Table 2. In addition, our previous results showed that the amount of GAGs and bound water in bone matrix was significantly reduced in KO mice compared with WT mice [33]. These *in situ* results indicate that coupling with water biglycan is apparently one of PGs in bone mineral matrix that plays a key role in sustaining the toughness of bone. Our recent study demonstrates that loss of GAGs may significantly reduce the tissue-level toughness of bone and such effects are most likely induced by the associated loss of bound water in bone matrix [10], and the results show the similarity as that among the age-related ultrastructural changes [19,20,21,22], the loss of bound water in bone matrix with significant reduction of the toughness of bone [23,24,25,26,27]. Based on the aforementioned evidence, it could be conjectured that changes in matrix GAGs may contribute to the deterioration of bone quality.

**Figure 3.**
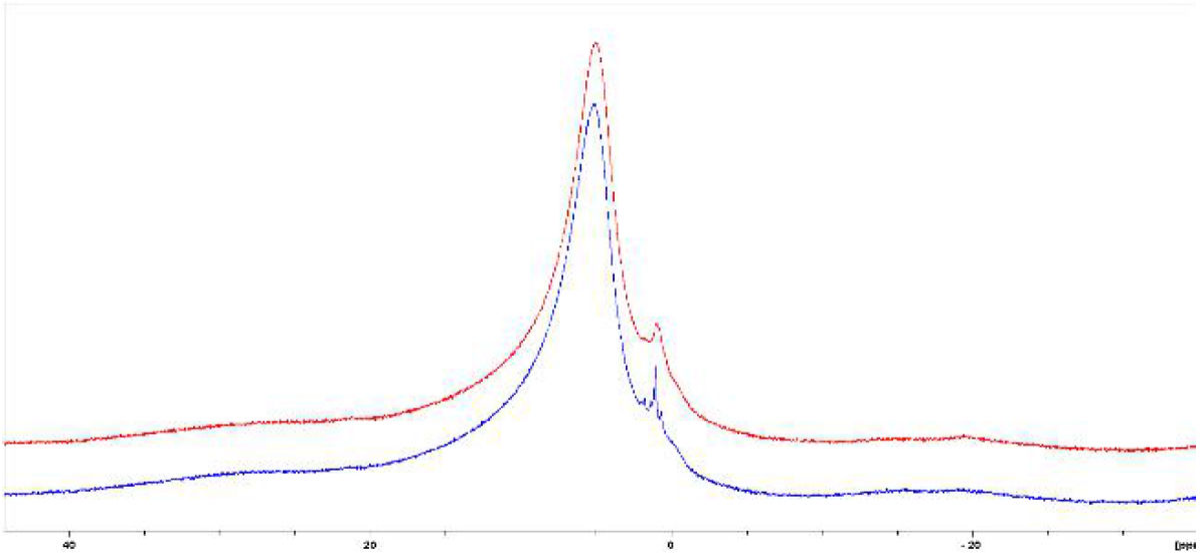
^1^H spectra of mice leg bones for the same samples as in Figure 1, WT and KO. The top one (red), and the bottom one (blue) are the KO and WT, respectively, measured by high-field (400 MHz) proton NMR with MAS probe.

Figure 3 demonstrates ^1^H NMR spectra of the intact mice bone tissues obtained from solid-state MAS measurement that shows two peaks as indicates by Singh [28], one peak at 5.2 ppm comes from water (bound) in bone matrix and second one at 1.4 ppm is assigned to hydroxyl ion (OH−) attached to Ca^++^ surface. Kaflak-Hachulska et al., [29] assigned this peak as hydroxide ion in a unique hydrogen-bonded state in carbonated apatite. Other studies by Wilson et al. [2,30] suggested that this peak might come from organic matrix [2,30]. But due to insufficient resolution and heterogeneous environment in their studies of bone, this signal was not clearly identified. So we cannot say conclusively that OH^−^ at 1.4 ppm is coming from organic matrix or attached to Ca^2+^ [28]. But all studies confirm that it was coming from hydroxide ion (OH^−^). It is observed that WT spectrum has better peak resolution at 1.4 ppm than KO one. In study it is found that the average mineral component of calcium hydroxyapatite (Ca_10_(OH)_2_(PO_4_)_6_) is reduced compared with the control group.

## 4. Conclusions

This is the first attempt that the porosity, and the bound water have been measured non-invasively and non-destructively in biglyan induced mouse bone using a NMR approach. The porosity, and bound water pattern differences between WT and KO groups are observed. These differences can be used to predict and correlate with the bone mechanical properties.

Studying mice with targeted Bgn disruption has revealed the involvement of Bgn in the regulation of postnatal skeletal growth. Bgn deficiency may lead to bone porosity increase, bone bound water decrease and bone biomechanical strength changes. This study has investigated the specific role of Bgn in retaining bound water in bone matrix and maintaining bone toughness using a Bgn-deficient mouse model. Biochemical, biophysical, and biomechanical experiments performed to determine the amount of total GAGs, CS and bound water, and their effects on the tissue-level toughness of bone. The coupling effect of proteoglycans and bound water in maintaining bone toughness has been shown in human bone during the aging process [31,32]. To examine the effects of Bgn deficiency on the amount of bound water, cortical bone lysates of WT and Bgn KO mice were analyzed by low-field NMR measurements. Results showed that a significant reduction in bound water in bone matrix [33]. Additionally, in study by high-field MAS NMR it is found that the average mineral component of calcium hydroxyapatite (Ca_10_(OH)_2_(PO_4_)_6_) is reduced compared with the control group. It may be also related to the bone toughness reduction after Bgn KO mice group compared with control group. The techniques for the investigation have been developed and the further studies are needed.

## Figures and Tables

**Table 1.** Low-Field NMR Results for the % of porosity of mice.

Sample	Sample weight (g)	Volume (air-H20)	Porosity %
WT1	0.0489	0.0165	27.88%
WT2	0.0504	0.0206	33.81%
WT3	0.0412	0.0171	21.33%
WT4	0.0437	0.0194	40.99%
WT5	0.0402	0.0250	51.01%
WT6	0.0388	0.0283	21.07%
WT7	0.0382	0.0263	38.18%
		Average	33.47%±10.94%
KO1	0.0428	0.0123	33.44%
KO2	0.0412	0.0231	38.49%
KO3	0.0440	0.0188	37.78%
KO4	0.0372	0.0173	56.12%
KO5	0.0393	0.0159	52.93%
KO6	0.0444	0.0168	55.48%
KO7	0.0340	0.0189	55.20%
		Average	47.06%±9.98%

**Table 2.** Bound water of mice determined by NMR measurements.

	Weight	A1 (solid)	A2 (bound)	A2/A1
WT1	0.0489	0.0341	0.0208	0.6100
WT2	0.0504	0.0354	0.0211	0.5960
WT3	0.0412	0.0404	0.0184	0.4554
WT4	0.0437	0.0426	0.0233	0.5475
WT5	0.0402	0.0329	0.0194	0.5896
WT6	0.0388	0.0348	0.0182	0.5232
WT7	0.0382	0.0321	0.0177	0.5524
AVG				0.5534±0.0530
KO1	0.0428	0.0388	0.0171	0.4407
KO2	0.0412	0.0361	0.0177	0.4903
KO3	0.0440	0.0369	0.0180	0.4878
KO4	0.0372	0.0444	0.0232	0.5226
KO5	0.0393	0.0433	0.0237	0.5469
KO6	0.0444	0.0501	0.0237	0.4727
KO7	0.0340	0.0467	0.0200	0.4283
			AVG	0.4842±0.0421
